# Elements of General Pathology; a Practical Treatise on the Causes, Forms, Symptoms and Results of Disease

**Published:** 1848-05

**Authors:** 


*Elements of General Pathology: a practical treatise on the causes, forms, symptoms, and results of disease.* By Alfred Stillé, M. D., Lecturer on Pathology and the Practice of Medicine; Member of the Medical Society of Observation of Paris; Fellow of the Philadelphia College of Physicians, etc. 12mo. pp. 483. Lindsay & Blakiston. Philadelphia: 1848.

This is one of a series of works which the enterprising publishers are bringing out for the use of medical practitioners and students—manuals, or hand-books, on the various branches of medical science usually taught in our medical schools.

The author of the present volume, although yet far from being in "the sear and yellow leaf," is advantageously known to the profession in the United States as an officer of the late National Medical Convention, and as a well read physician and ready writer. To the opportunities afforded in this country for studying his profession, he has added those of Europe, and particularly of the French metropolis, where his mind has manifestly received its bent. With him Louis is prophet, and numerical medicine is his creed; and those who are not of the same household of faith receive little notice from his pen.

The opinion of the author as to what has been written on the subject of the present work by British and American physicians, may be inferred from the following remarks contained in the Preface:

"English Medical Literature has hitherto possessed no work exclusively devoted to General Pathology. In nearly all of the systematic treatises and dictionaries of medicine in our language, this subject is either discussed theoretically and imperfectly, or is altogether unnoticed." The object of the author is "to supply the deficiency which is thus disclosed in our medical literature;" and whether the more exclusive character claimed for his production has contributed to render it less theoretical and less imperfect than its predecessors, will be variously considered by its readers. It is not our purpose, in the present notice, to enter upon this discussion, nor to engage at all in a critical examination of the author's views and arguments, but simply to commend the work to the notice of our readers as the production of an intelligent writer on an interesting branch of medical science, the study of which is far too much neglected by students and practitioners generally.

